# Frequencies of VKORC1-1639G>A and rs397509427 in Patients on Warfarin and Healthy Syrian Subjects

**DOI:** 10.1155/2023/8898922

**Published:** 2023-11-23

**Authors:** Yara Altawil, Lama A. Youssef

**Affiliations:** ^1^Department of Pharmaceutics and Pharmaceutical Technology, Program of Clinical and Hospital Pharmacy, Faculty of Pharmacy, Damascus University, Damascus, Syria; ^2^National Commission for Biotechnology, Damascus, Syria

## Abstract

**Background:**

Vitamin K epoxide reductase complex subunit 1 (*VKORC1*) gene encodes a key enzyme with multiple cellular activities, namely, the reduction of vitamin K to its active form. *VKORC1*-1639G>A (rs9923231) is a common single nucleotide polymorphism with a crucial impact on warfarin dosing and possibly other physiological functions. This study aimed at investigating the frequencies of *VKORC1*-1639G>A alleles and genotypes in Syrian healthy subjects and patients on warfarin for different indications.

**Methods:**

A total of 138 individuals were enrolled in this cross-sectional study. Genomic DNA was extracted from both patients on warfarin and healthy subjects, and polymerase chain reaction (PCR) specific amplicons were genotyped via standard sequencing which also allowed the detection of rs397509427. Comparisons of -1639G>A frequency with other populations were drawn.

**Results:**

Of 94 patients on warfarin, 53 (56.38%) were with idiopathic venous thromboembolism (VTE). Despite comparable frequencies of the -1639A allele (47% and 50%), the AA and GA genotypes were at disparate frequencies of 93.2% versus 79.8% in the healthy subjects (*n* = 44) versus patients on warfarin, respectively. Carriers of the GG genotype were at a four-fold increased risk of VTE in comparison with those of the AA and GA genotypes (odds ratio (OR) = 4, 95% CI = 1.105 − 13.6, *P* = 0.0469). All study subjects were wild-type for the rs397509427 variant.

**Conclusions:**

Our results prove a high -1639A prevalence in Syrian healthy subjects and patients on warfarin at frequencies comparable to other Mediterranean and Middle Eastern populations. The A allele carriers are at a lower VTE risk, whereas a global prevalence gradient of the G allele is suggested to be associated with VTE risk.

## 1. Background

Vitamin K-dependent proteins (VKDPs) have crucial roles in several physiological functions, including blood coagulation, arterial calcification prevention, prenatal heart development, calcium regulation, bone homeostasis, and multiple cellular activities such as growth and inflammatory response [1, 2]. A posttranslational modification step, mediated by gamma-glutamyl carboxylase (GGCX) enzyme that utilizes vitamin K hydroquinone (KH2) as a cofactor, must take place in order to activate VKDPs [1]. Concomitantly, the KH_2_ is converted to its oxidized form vitamin K 2,3-epoxide (KO), which induces its recycling to the reduced form to compensate for the rapid consumption of KH_2_ [3]. Vitamin K epoxide reductase (VKOR) controls the rate of the carboxylation reaction by exclusively reducing KO to vitamin K and also assisting the next step of vitamin K to KH_2_ conversion in this vitamin K cycle [4].

Vitamin K epoxide reductase complex subunit 1 (*VKORC1*) gene that encodes the VKOR enzyme is located on chromosome 16 [5]. Of the several single nucleotide polymorphisms (SNPs) identified in the *VKORC1* gene, “-1639G>A” is the most prominent due to its impact on dosing of warfarin, which exerts its effect by inhibiting VKOR, and subsequently impeding the activation of the coagulation factors II, VII, IX, X [4–6]. The -1639G>A “rs9923231” is located in the promoter region of *VKORC1* gene and the presence of the A allele reduces gene expression leading to fewer mRNA transcripts and eventually fewer copies of the enzyme [7]. Thus, the carriers of the AA genotype must have their therapeutic doses reduced by 70%, according to the drug label released in 2007 by the Food and Drug Administration (FDA) [6]. Owing to the *VKORC1*-1639G>A significance, previous studies attempted to investigate a plausible association between it and other diseases such as stroke, venous thromboembolism (VTE), cardiovascular diseases, and osteoporosis [8–10].

A plethora of studies on the frequency of *VKORC1*-1639G>A among Caucasians and other ethnicities (such as Chinese and Africans) has created a wealth of information that aided in the generation of warfarin dosing algorithms [11]; however, studies on the prevalence of the *VKORC1*-1639G>A allele in Middle Eastern populations, including Syrians, are relatively scarce. Although Syrians' genetic makeup is predicted to be similar to that of other populations in the Middle East and Mediterranean basin, nonetheless, the population of Syria exhibits an extremely diverse ethnic composition, consisting of a majority of Arab descents, as well as minorities of Kurds, Assyrians, Armenians, Turkmens, and others. Moreover, Syria has always been a mosaic of diverse faiths and “ethnoreligious” groups; although approximately 90% of all Syrians are Muslim and almost all the rest are Christians, Syrians from both religions are divided up into many sects. Therefore, it is vital to determine the genetic composition of such an ethnically, religiously, and culturally diverse population, as a prerequisite for the implementation of pharmacogenetic-guided dosing approach. Hence, this study aimed at investigating the frequency of *VKORC1*-1639G>A in Syrian healthy volunteers as well as patients on warfarin for different indications, including idiopathic VTE. Comparisons of -1639G>A frequency with other populations were drawn, and a proposed hypothesis of association between the G allele prevalence with increased VTE risk globally was investigated.

## 2. Methods

### 2.1. Study Subjects

A total of 138 unrelated Syrian individuals were enrolled in the study from three major governorates (Damascus, Damascus Countryside, and Homs) from October 2018 to September 2020. Ninety-four outpatients treated with warfarin for different indications, including 53 (56.38%) with idiopathic VTE, were recruited from several hospitals and medical laboratories. The exact number of patients for every indication is shown in Supplementary File 1. Of the 94 patients, 54 were males and 40 females, with a mean age ± standard deviation (SD) of 47 ± 14.48. Forty-four healthy unrelated subjects matched with the patients in terms of age and gender (20 males and 24 females, with a mean age ± SD of 49.52 ± 18.46) were enrolled for the purpose of allele and genotype frequency comparison. Informed consents were obtained from all participants and the legal guardian of one 17-year-old male patient. This cross-sectional study was approved by the Scientific Research Bioethics Committee at the Faculty of Pharmacy, Damascus University; and all methods were performed in accordance with its regulation.

### 2.2. Genotyping

Genomic DNA was extracted from peripheral blood samples using a solution-based DNA isolation kit (Wizard® Genomic DNA Purification Kit, Promega®, USA) according to the manufacturer's protocol. -1639G>A genotyping was performed via standard sequencing of specific polymerase chain reaction (PCR) products using specific primers. The amplicon's length allowed for the detection of another SNP (rs397509427), which is recognized by the insertion of cytosine nucleotide [12]. The reactions contained 30-100 ng of gDNA, 20 pmol of each primer, and 20 *μ*L of master mix (OnePCR™, GeneDirex®, Taiwan); thereafter, the volume was completed with nuclease-free water to 40 *μ*L.

A pair of primers previously published in relevant literature [13, 14] for the amplification of *VKORC1*-1639G>A containing region was tried at first. Then, we designed another pair using Geneious Pro 4.8.4 (Biomatters®, Auckland, New Zealand). The specificity of our de novo designed forward primer (5′-GCCAGCAGGAGAGGGAAATATCA-3′) and reverse primer (5′-TGGTGTCACCAAGACGCTAGAC-3′) was confirmed using BLAST (NCBI, USA) and MFEPrimer 3.1(iGeneTech®, China) and synthesized at Macrogen® Inc. (Seoul, South Korea). The cycling profile is summarized in Table 1.

All reactions yielded the expected product that was confirmed by the appearance of a single band of 457 base pair (pb) length via ethidium bromide-stained 1.5% agarose gel electrophoresis. Amplicons sequencing was conducted at Macrogen®, using the ABI PRISM® BigDye™ Terminator Cycle Sequencing Kit with an ABI PRISM® 3730XI DNA Analyzer, after an enzymatic clean-up method using EnzSAP™ PCR Clean-up Reagent. A representative gel image with representative sequencing chromatograms are shown in Supplementary File 2.

### 2.3. Statistical Analysis

Statistical analysis was performed using SPSS® (version 25) and GraphPad Prism® (Version 8) software. The observed frequencies of *VKORC1*-1639G>A genotypes were compared to the expected frequencies from the Hardy–Weinberg equilibrium (HWE) using the chi-square goodness of fit test. The difference between patients' and healthy subjects' genotypes was assessed using the chi-square test, whereas Fisher's exact test was applied to compare the frequencies of the A carriers between VTE patients and healthy subjects and estimate the odds ratio with its corresponding 95% confidence intervals (CI). A *P* value less than 0.05 was considered to be statistically significant in all performed tests.

## 3. Results

Forty-four healthy subjects and 97 patients were enrolled; however, three patients were nonassessable due to inadequate blood samples (*n* = 2) and unsuccessful genotyping (*n* = 1). Thus, the final number of assessable patients was 94. We screened for both rs9923231 (-1639G>A) and rs397509427; however, only the -1639G>A was identified in the study subjects. The heterozygous genotype (GA) constituted the highest frequency genotype in both study groups, and the A allele carriers (GA+AA) represented 79.8% and 93.2% of the patients and healthy subjects, respectively (*P* = 0.049). Genotypes and allele frequencies for study groups are presented in Table 2. Both groups exhibited a significant deviation from HWE with *P* values of 0.002 and less than 0.001 for the patients and healthy subjects, respectively. Expected values from HWE are shown in Supplementary File 3.

### 3.1. Association between the -1639G>A SNP and VTE Risk

There was a significant difference between the healthy and patients' groups in terms of the genotype distribution (*P* = 0.0415). Therefore, a comparison was drawn between the healthy subjects and the patients diagnosed with idiopathic (unprovoked) VTE (*n* = 53). Carriers of GG genotype had shown a 4-fold increased risk of VTE compared to the A allele carriers (GA+AA) (odds ratio (OR) = 4, 95% CI = 1.105 − 13.6, *P* = 0.0469) as summarized in Table 3. Nevertheless, no statistically significant difference (*P* = 0.29) was noticed in the GG genotype frequencies (6.8% and 16.2%, respectively) in healthy subjects versus patients (*n* = 37) on warfarin for other than VTE indications (i.e., heart valve replacement, ischemic stroke, and atrial fibrillation).

## 4. Discussion

**Figure 1:**
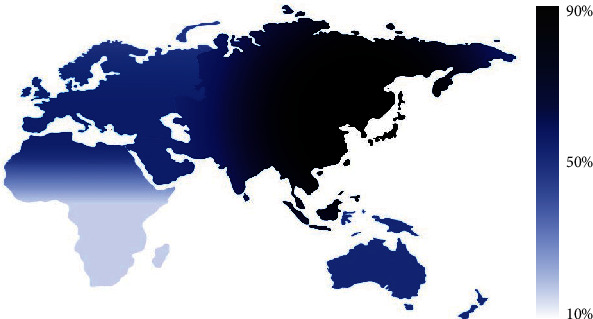
The frequency gradient of *VKORC1*-1639A in the continents of Europe, Asia, and Africa as reported by the studies cited in Table 4. The highest frequency has been reported in Far-Eastern Asian countries (i.e., China and Japan) where it reaches 90% (dark navy), whilst the least frequency is seen in South Africa with a frequency of about 10% (light blue).

-1639G>A SNP in *VKORC1* gene has been linked to a wide variety of diseases in addition to their crucial role in warfarin dosing, in different populations and ethnicities [2]. This study is the first, to the best of our knowledge, that investigated the frequency of *VKORC1*-1639G>A in Syrians, both patients on warfarin and healthy volunteers. Our findings demonstrate a high prevalence of the -1639A variant allele in the study populations (47% and 50% in patients and healthy Syrians, respectively). These frequencies were comparable to the frequencies in neighboring populations and the Mediterranean basin populations, whilst greater differences were evident with those of South-African and East-Asian populations [15]. The frequencies of -1639A allele in various populations and countries are summarized in Table 4. The A allele seems to follow a frequency gradient, in which the highest intensity is seen in East Asian populations e.g. Japanese and Chinese, where it is thought to be originated. It starts to decrease towards the southwest of the old-world map, where it becomes relatively low in the south of the African continent (Figure 1).

On the other hand, North and South Americas have proved a discrepancy in the A allele frequency according to the ethnicities of the individuals: Caucasians, Latin Americans, Asians, or Africans [15]. Syria and the Levantine countries in general have both alleles nearly equally prevalent, which could be explained by its diverse genetic makeup of the populations. Its pivotal location at the heart of the old world, between the eastern Mediterranean coast and the Euphrates Valley, has placed Syria at the crossroads of the Eurasian silk and spice trade routes, active from the second century BCE until the mid-15th century. Additionally, wars and multihuman migration waves throughout the history of the Levant have resulted in remarkable ethnic, cultural, and genetic diversity [31].

The heterozygous genotype GA reached higher percentages than expected, based on HWE, in both study groups. This deviation from HWE is less likely to be attributed to genotyping error as standard sequencing was used for the genotype assignment. However, a possible explanation of this deviance might stem from the recent Syrian war that caused dislocations and displacement of millions of Syrians who have fled the country throughout the ten-year War. It is speculated that these tragic circumstances have caused a demographic skewness in the Syrian population causing a subtle kind of stratification [32–34].

Another probable reason for such a deviation from HWE stems from the cultural and religious norms, including the high rate of consanguineous marriages, in this Middle Eastern population. Nevertheless, all enrolled subjects in this study were unrelated. A third likable justification is the small sample size since one of the basic assumptions of HWE is an infinitely large population where genetic drift can be ignored. Therefore, a large sample size is preferred in order to exclude selection bias and make the sample well-representative to the community, although this is less likely since the size of our study population is comparable to those in several other frequency studies [17, 23, 33].

Our patients and healthy subjects had similar allele frequencies. To the contrary, a significantly different genotype distribution between healthy subjects and patients with VTE was evident, suggesting an association between the GG genotype with thrombophilia. VTE is a multifactorial disease with a complicated interaction between genetic and environmental factors, and it is estimated that half of the cases occurred without identifiable risk factors [35]. In our study, only unprovoked VTE patients were compared with the healthy subject to negate any other evident causes of the disease such as surgery or pregnancy. The carriers of the A allele comprising genotypes (GA+AA) had a lower risk (75%) for developing VTE than the GG genotype carriers which could be ascribed to the decrease in gene expression of the VKORC1, leading to a reduced protein synthesis (by 44% in homozygotes). The decline in VKORC1 enzyme might affect both the activation status and plasma levels of vitamin K-dependent coagulation factors [9]. Carrying the A allele can be considered as a lifelong administration of anticoagulant at a low dose to prevent or alleviate thrombotic accidents.

A plethora of genetic mutations and polymorphisms are known to be risk factors (i.e., deficiencies of proteins C, S, and antithrombin III; factor V Leiden (FVL); prothrombin (PT) 20210A mutation; and non-O blood groups) for developing VTE [36]. Nevertheless, interindividual variability could be partially explained by these factors; as only 30% of unprovoked VTE cases have been linked to a well-characterized genetic risk factor. Furthermore, the interethnic variations in VTE incidence manifest as a greater dilemma. Although the highest incidence rates of thrombophilia or hypercoagulability (i.e., VTE) have been reported in Africans and African descendants followed by Caucasians and Asians, respectively, the frequencies of the aforementioned familiar genetic risk factors do not correlate with such between-ethnicity disparity in VTE prevalence. For instance, FVL and PT mutations are well-known prothrombotic factors, with FVL being the most common hereditary thrombophilia; however, FVL and PT mutations have the least frequencies in Africa. Furthermore, the frequencies of protein C & S deficiencies in Africa and Europe are comparable. Remarkably, the frequencies of the *VKORC1*-1639G risk allele are reasonably correlated with VTE incidence around the world, where its highest frequency recorded in Africa (88%) followed by Europe (58%) and the least in Asia (10%) [37–39]. The main limitation of this study is the sample size. Therefore, further prospective studies on larger number of subjects are called for to prove or refute the causality association between *VKORC1*-1639GG genotype and VTE incidence.

## 5. Conclusions

This study proves a high prevalence of the *VKORC1*-1639A allele in Syrians, with almost equal frequencies in both patients on warfarin (50%) and healthy subjects (47%). Our findings shed light on the frequency gradient of this allele across the globe. It also suggests a possible protective effect for the -1639A allele against VTE. Moreover, our study may help reveal another piece of the complicated VTE genetic predisposition puzzle.

## Figures and Tables

**Table 1:** The cycling profile of the newly designed primers for VKORC1-1639G>A.

Step	Temperature	Duration	Cycles
Initial denaturation	94°C	5 min	1
Denaturation	94°C	30 sec	35
Annealing	60°C	30 sec	
Extension	72°C	60 sec	
Final extension	72°C	10 min	1

**Table 2:** Frequencies of *VKORC1-1639G>A* genotypes and alleles among study groups.

*VKORC1-1639G>A*		Patients (*n* = 94)	Healthy subjects (*n* = 44)	*P* value	Total (*n* = 138)
		No. (%)	No. (%)		No. (%)
Genotype	GG	19 (20.2)	3 (6.8)	0.0415	22 (15.9)
	GA	62 (66)	38 (86.4)		100 (72.5)
	AA	13 (13.8)	3 (6.8)		16 (11.6)

Allele	G	0.53	0.5	0.698	0.52
	A	0.47	0.5		0.48

**Table 3:** Frequencies of *VKORC1*-1639G>A genotypes between healthy subjects and patients with unprovoked VTE.

Genotype	Unprovoked VTE (*n* = 53)	Healthy subjects (*n* = 44)	OR (95% CI)	*P* value
	No. (%)	No. (%)		
GG	12 (22.6)	3 (6.8)	4 (1.105-13.60)	0.0469
AA+GA	41 (77.4)	41 (93.2)	0.25 (0.07195-0.9047)	

**Table 4:** The frequency of -1639A allele in different populations.

Country (reference)	No. of individuals		Frequency of the -1639A	
	Patients	Healthy		
Syria (our study)	94	44	47.8	
Japan [16]	125	114	89.2%	93.9%
South Africa [17]	89	—	12%	
Germany [18]	91	—	39%	
Australia [19]	5408	—	40.3%	
Egypt [20]	100	—	44%	
Turkey [21]	200	—	44%	
Morocco [22]	217ǂ	—	46.1%	
Palestine/Gaza [23]	101	—	46.5%	
Italy [24]	264	—	49.8%	
China [25]	278	—	91.4%	
France [26]	—	222	42%	
Saudi Arabia [27]	—	131	42.7%	
United Arab Emirates [28]	—	117	49.6%	
Lebanon [29]	—	161	52.8%	
Iran [30]	—	126	55.6%	

ǂPatients are on acenocoumarol (another coumarin anticoagulant).

## Data Availability

The datasets generated and analysed during the current study are available in the ClinVar repository, with the accession number SCV003836537.

## Abbreviations

bp: Base pairs
CI: Confidence intervals
FVL: Factor V Leiden
FDA: Food and Drug Administration
GGCX: Gamma-glutamyl carboxylase
HWE: Hardy–Weinberg equilibrium
KO: Vitamin K 2,3-epoxide
KH2: Vitamin K hydroquinone
OR: Odds ratio
PCR: Polymerase chain reaction
PT: Prothrombin
SD: Standard deviation
SNP: Single nucleotide polymorphism
VKDPs: Vitamin K-dependent proteins
VKOR: Vitamin K epoxide reductase
VKORC1: Vitamin K epoxide reductase complex subunit 1
VTE: Venous thromboembolism.

## Supplementary Materials

Supplementary 1 Includes Table S1. that summarizes classification of patients (*n* = 94) according to warfarin indications.

Supplementary 2 Includes Figure S2.1. that provides a representative gel electrophoresis of PCR products encompassing the *VKORC1*-1639G>A polymorphism, Figure S2.2. that presents sequencing chromatograms showing examples of wild-type GG (A); homozygote AA (B), and heterozygote GA (C) of the VKORC1-1639G>A polymorphism, and Figure S2.3. that displays a sequencing chromatogram for VKORC1 rs397509427.

Supplementary 3 Includes Table S2. entitled “Frequencies of the observed VKORC1-1639G>A genotypes and those expected from Hardy-Weinberg Equilibrium (HWE) in patients on warfarin and healthy subjects.”

## References

[1] Chatron N., Hammed A., Benoît E., Lattard V. (2019). Structural insights into phylloquinone (vitamin K1), menaquinone (MK4, MK7), and menadione (vitamin K3) binding to VKORC1. *Nutrients*.

[2] Caspers M., Czogalla K., Liphardt K. (2015). Two enzymes catalyze vitamin K 2,3-epoxide reductase activity in mouse: VKORC1 is highly expressed in exocrine tissues while VKORC1L1 is highly expressed in brain. *Thrombosis Research*.

[3] Tie J. K., Stafford D. W. (2017). Functional study of the vitamin K cycle enzymes in live cells. *Methods in Enzymology*.

[4] Liu S., Li S., Shen G., Sukumar N., Krezel A., Li W. (2021). Structural basis of antagonizing the vitamin K catalytic cycle for anticoagulation. *Science*.

[5] Yang L., Ge W., Yu F., Zhu H., Zhu H. (2010). Impact of _VKORC1_ gene polymorphism on interindividual and interethnic warfarin dosage requirement -- A systematic review and meta analysis. *Thrombosis Research*.

[6] Eriksson N., Wadelius M. (2012). Prediction of warfarin dose: why, when and how?. *Pharmacogenomics*.

[7] Owen R. P., Gong L., Sagreiya H., Klein T. E., Altman R. B. (2010). VKORC1 pharmacogenomics summary. *Pharmacogenetics and Genomics*.

[8] Dubovyk Y. I., Harbuzova V. Y., Ataman A. V. (2016). G-1639A but Not C1173T _VKORC1_ Gene Polymorphism Is Related to Ischemic Stroke and Its Various Risk Factors in Ukrainian Population. *BioMed Research International*.

[9] Kumari B., Garg I., Rai C., Panjawani U., Bhuvnesh K., Srivastava S. (2019). Positive association of mutations in VKORC1 and CYP2C9 genes with venous thrombo-embolism (VTE) in Indian population: a case control study. *Journal of Genetic Engineering and Biotechnology Research*.

[10] He J., Xie H., Yan C., Sun Y., Xu Z., Zhang X. (2021). Genetic variation in VKORC1 and risk for osteoporosis. *Archives of Medical Research*.

[11] Tavares L. C., Marcatto L. R., Santos P. C. (2018). Genotype-guided warfarin therapy: current status. *Pharmacogenomics*.

[12] *rs397509427 RefSNP Report - db SNP-NCBI*.

[13] Sconce E. A., Khan T. I., Wynne H. A. (2005). The impact of CYP2C9 and VKORC1 genetic polymorphism and patient characteristics upon warfarin dose requirements: proposal for a new dosing regimen. *Blood*.

[14] Zhu J., Zhang W., Li Y. (2010). ARMS test for diagnosis ofCYP2C9andVKORC1mutation in patients with pulmonary embolism in Han Chinese. *Pharmacogenomics*.

[15] *rs9923231 RefSNP Report - dbSNP-NCBI*.

[16] Obayashi K., Nakamura K., Kawana J. (2006). VKORC1 gene variations are the major contributors of variation in warfarin dose in Japanese patients. *Clinical Pharmacology and Therapeutics*.

[17] Ndadza A., Cindi Z., Makambwa E. (2019). Warfarin dose and CYP2C gene cluster: an African ancestral-specific variant is a strong predictor of dose in Black South African patients. *OMICS: A Journal of Integrative Biology*.

[18] Luxembourg B., Schneider K., Sittinger K. (2011). Impact of pharmacokinetic (CYP2C9) and pharmacodynamic (VKORC1, F7, GGCX, CALU, EPHX1) gene variants on the initiation and maintenance phases of phenprocoumon therapy. *Thrombosis and Haemostasis*.

[19] Mostafa S., Kirkpatrick C., Byron K., Sheffield L. (2019). An analysis of allele, genotype and phenotype frequencies, actionable pharmacogenomic (PGx) variants and phenoconversion in 5408 Australian patients genotyped for CYP2D6, CYP2C19, CYP2C9 and VKORC1 genes. *Journal of Neural Transmission*.

[20] Selim T. E., Azzam H. A., Ghoneim H. R., Mohamed A. A., El Wakeel H., Abu Bakr H. M. (2018). Pharmacogenetic warfarin dosing algorithms: validity in Egyptian patients. *Acta Haematologica*.

[21] Kocael A., Eronat A., Tüzüner M. (2019). Interpretation of the effect of CYP2C9, VKORC1 and CYP4F2 variants on warfarin dosing adjustment in Turkey. *Molecular Biology Reports*.

[22] Elkhazraji A., Bouaiti E. A., Boulahyaoui H. (2018). Effect of *CYP2C9*, *VKORC1*, *CYP4F2*, and *GGCX* gene variants and patient characteristics on acenocoumarol maintenance dose: proposal for a dosing algorithm for Moroccan patients. *Drug Discoveries & Therapeutics*.

[23] Ayesh B. M., Abu Shaaban A. S., Abed A. A. (2018). Evaluation of *CYP2C9-* and *VKORC1-*based pharmacogenetic algorithm for warfarin dose in Gaza-Palestine. *Future Science OA*.

[24] Mazzaccara C., Conti V., Liguori R. (2013). Warfarin anticoagulant therapy: a southern Italy pharmacogenetics-based dosing model. *PLoS One*.

[25] Yan X., Yang F., Zhou H. (2015). Effects of VKORC1 genetic polymorphisms on warfarin maintenance dose requirement in a Chinese Han population. *Medical Science Monitor: International Medical Journal of Experimental and Clinical Research*.

[26] Bodin L., Verstuyft C., Tregouet D. A. (2005). Cytochrome P450 2C9 (CYP2C9) and vitamin K epoxide reductase (VKORC1) genotypes as determinants of acenocoumarol sensitivity. *Blood*.

[27] Alzahrani A. M., Ragia G., Hanieh H., Manolopoulos V. G. (2013). Genotyping of _CYP2C9_ and _VKORC1_ in the Arabic Population of Al-Ahsa, Saudi Arabia. *BioMed Research International*.

[28] Al-Jaibeji H. S., John A., Al-Gazali L. (2016). Allele and genotype frequencies of the two single nucleotide polymorphisms in the VKORC1 gene that are most important for warfarin treatment among Emiratis. *Hamdan Medical Journal*.

[29] Djaffar-Jureidini I., Chamseddine N., Keleshian S., Naoufal R., Zahed L., Hakime N. (2011). Pharmacogenetics of coumarin dosing: prevalence of CYP2C9 and VKORC1 polymorphisms in the lebanese population. *Genetic Testing and Molecular Biomarkers*.

[30] Azarpira N., Namazi S., Hendijani F., Banan M., Darai M. (2010). Investigation of allele and genotype frequencies of _CYP2C9, CYP2C19_ and _VKORC1_ in Iran. *Pharmacological Reports*.

[31] *About the Silk Roads|Silk Roads Programme*.

[32] Qayyum A., Najmi M. H., Mansoor Q. (2018). Frequency of common VKORC1 polymorphisms and their impact on warfarin dose requirement in Pakistani population. *Clinical and Applied Thrombosis/Hemostasis*.

[33] Wigginton J. E., Cutler D. J., Abecasis G. R. (2005). A note on exact tests of Hardy-Weinberg equilibrium. *American Journal of Human Genetics*.

[34] Zintzaras E. (2010). Impact of Hardy-Weinberg equilibrium deviation on allele-based risk effect of genetic association studies and meta-analysis. *European Journal of Epidemiology*.

[35] Benincasa G., Costa D., Infante T., Lucchese R., Donatelli F., Napoli C. (2019). Interplay between genetics and epigenetics in modulating the risk of venous thromboembolism: a new challenge for personalized therapy. *Thrombosis Research*.

[36] Rosendaal F. R., Reitsma P. H. (2009). Genetics of venous thrombosis. *Journal of Thrombosis and Haemostasis*.

[37] Crous-Bou M., Harrington L. B., Kabrhel C. (2016). Environmental and genetic risk factors associated with venous thromboembolism. *Seminars in Thrombosis and Hemostasis*.

[38] Scott S. A., Khasawneh R., Peter I., Kornreich R., Desnick R. J. (2010). CombinedCYP2C9,VKORC1andCYP4F2frequencies among racial and ethnic groups. *Pharmacogenomics*.

[39] Goldhaber S. Z. (2014). Race and venous Thromboembolism. *Circulation*.

