# Supplementary material for: Frequencies of VKORC1-1639G>A and rs397509427 in Patients on Warfarin and Healthy Syrian Subjects
Source: Cardiovasc Ther. 2023 Nov 23;2023:8898922. doi: 10.1155/2023/8898922 (PMC10689069; doi:10.1155/2023/8898922)
Supplement: Supplementary 1 — Includes Table S1. that summarizes classification of patients (n = 94) according to warfarin indications. [file 8898922.f1.pdf]

**Table S1. Classification of patients according to warfarin indications (n=94)**		
**Indications**	**No. of patients**	**Percentage**
VTE (DVT and/or PE)	57	60.6%
HVR	30	31.9%
Ischemic Stroke	4	4.3%
Atrial Fibrillation	3	3.2%
VTE: venous thromboembolism, DVT: deep vein thrombosis, PE: pulmonary embolism, HVR: heart valve replacement.		
