# Supplementary material for: Frequencies of VKORC1-1639G>A and rs397509427 in Patients on Warfarin and Healthy Syrian Subjects
Source: Cardiovasc Ther. 2023 Nov 23;2023:8898922. doi: 10.1155/2023/8898922 (PMC10689069; doi:10.1155/2023/8898922)
Supplement: Supplementary 2 — Includes Figure S2.1. that provides a representative gel electrophoresis of PCR products encompassing the VKORC1-1639G>A polymorphism, Figure S2.2. that presents sequencing chromatograms showing examples of wild-type GG (A); homozygote AA (B), and heterozygote GA (C) of the VKORC1-1639G>A polymorphism, and Figure S2.3. that displays a sequencing chromatogram for VKORC1 rs397509427. [file 8898922.f2.pdf]

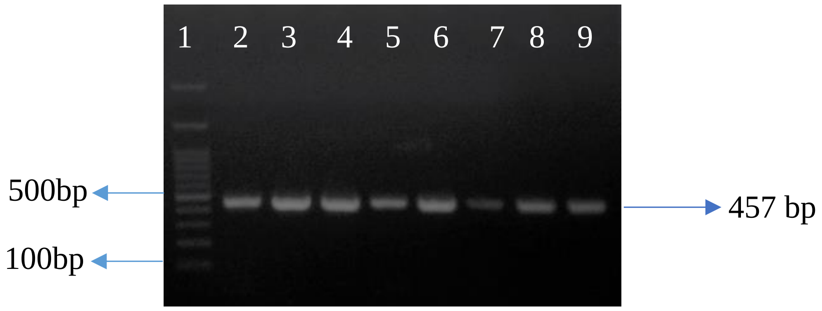

Figure S2.1. A representative gel electrophoresis of PCR products encompassing the VKORC1 -1639G>A polymorphism.

Lane 1, a 100 base-pair DNA marker ladder.

Lanes 2 to 9, PCR products with the expected length of 457bp representing eight subjects.

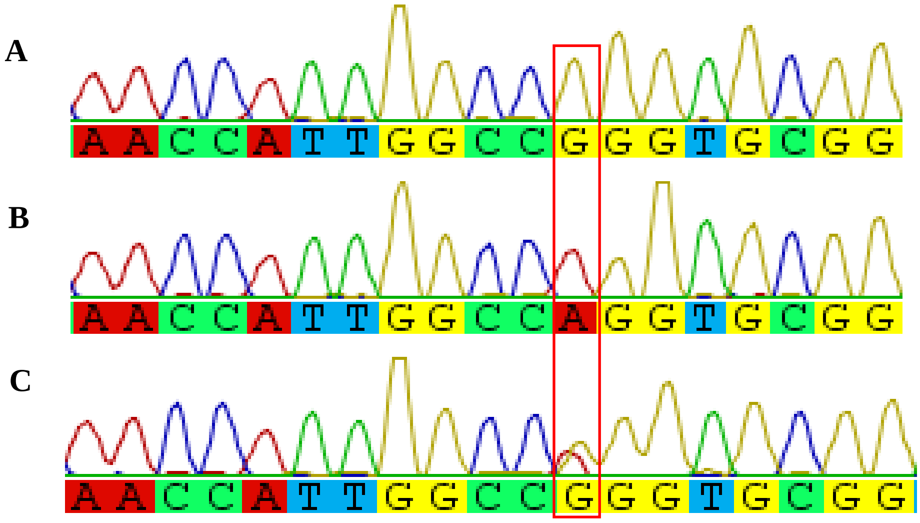

Figure S2.2. Sequencing chromatograms showing examples of wild-type GG (A); homozygote AA (B), and heterozygote GA (C) of the VKORC1 -1639G>A polymorphism.

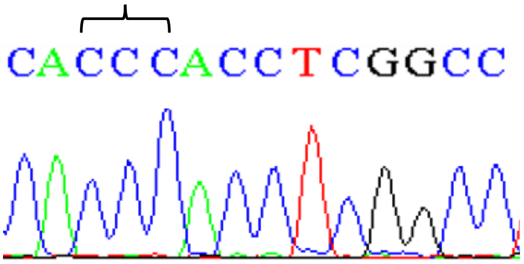

Figure S2.3. Sequencing chromatogram for VKORC1 rs397509427.

All samples in our study were wild-type and did not have the insertion of a fourth C nucleotide
