# Supplementary material for: Frequencies of VKORC1-1639G>A and rs397509427 in Patients on Warfarin and Healthy Syrian Subjects
Source: Cardiovasc Ther. 2023 Nov 23;2023:8898922. doi: 10.1155/2023/8898922 (PMC10689069; doi:10.1155/2023/8898922)
Supplement: Supplementary 3 — Includes Table S2. entitled “Frequencies of the observed VKORC1-1639G>A genotypes and those expected from Hardy-Weinberg Equilibrium (HWE) in patients on warfarin and healthy subjects.” [file 8898922.f3.pdf]

Table S2. Frequencies of the observed VKORC1-1639G>A genotypes and those expected from Hardy-Weinberg Equilibrium (HWE) in patients on warfarin and healthy subjects.

Genotype	Patients on warfarin (n=94)			Healthy Subjects (n=44)		
	observed	expected	HWE *P* value	observed	expected	HWE *P* value
**GG**	19	26.6	0.002	3	11	<0.001
**GA**	62	46.8		38	22	
**AA**	13	20.6		3	11	
